# Non-celiac wheat sensitivity: rationality and irrationality of a gluten-free diet in individuals affected with non-celiac disease: a review

**DOI:** 10.1186/s12876-020-01568-6

**Published:** 2021-01-06

**Authors:** Consolato Sergi, Vincenzo Villanacci, Antonio Carroccio

**Affiliations:** 1 Department of Laboratory Medicine and Pathology, Stollery Children’s Hospital, University of Alberta, 8440 112 St., Edmonton, AB T6G 2B7 Canada; 2 Institute of Pathology, ASST-Spedali Civili, Brescia, Italy; 3 Internal Medicine Unit, “V Cervello Hospital”, Department of Health Promotion Sciences, Maternal and Infant Care, Internal Medicine and Medical Specialties (PROMISE), University of Palermo, 90129 Palermo, Italy

**Keywords:** Celiac disease, Duodenum, Wheat, Allergy, Irritable bowel syndrome

## Abstract

Non-celiac gluten or wheat sensitivity (NCWS) is a “clinical entity induced by the ingestion of wheat leading to intestinal and/or extraintestinal symptoms that improve once the wheat-containing foodstuff is removed from the diet, and celiac disease and wheat allergy have been excluded”. This mostly accepted definition raises several points that remain controversial on this condition. In the present review, the authors summarize the most recent advances in the clinic and research on NCWS through an accurate analysis of different studies. We screened PubMed, Medline, Embase, and Scopus using the keywords “non-celiac gluten sensitivity”, “non-celiac wheat sensitivity”, and “diagnosis”. We would like to emphasize two main points, including (A) the controversial clinical and etiological aspects in different trials and experiences with particular attention to the Salerno criteria for the diagnosis of NCWS and (B) the histological aspects. The etiology of NCWS remains controversial, and the relationship with irritable bowel syndrome is obscure. Histologically, the duodenal mucosa may show a variable pattern from unremarkable to a slight increase in the number of T lymphocytes in the superficial epithelium of villi. The endorsement of this disease is based on a positive response to a gluten-free diet for a limited period, followed by the reappearance of symptoms after gluten challenge. The Salerno expert criteria may help to diagnose NCWS accurately. Social media and inaccurate interpretation of websites may jeopardize the diagnostic process if individuals self-label as gluten intolerant.

## Background

For more than 40 years, the polymorphic spectrum of presentation in clinical symptoms, laboratory data and histological characteristics of celiac disease has been compared to a “chameleon” [1, 2], and gastroenterologists seek refuge in further diagnostic tests or more complicated levels of genetic diagnosis. If all these test results are negative, it is suggested to indicate that the patient may have so-called non-celiac wheat sensitivity (NCWS) (alternatively labeled non-celiac gluten sensitivity by some authors). In this review, the authors show recent advances and problems in the clinic and research on NCWS and report their experience in facing clinical and histological doubts in this field.

### Methodology of the search strategy

We performed a literature search in PubMed (http://www.ncbi.nlm.nih.gov/pubmed/), Medline, Embase, Scopus, and Google Scholar (up to April 30, 2020) using the following search terms in the title and abstract: ‘Non-celiac gluten sensitivity’ or ‘Non-celiac wheat sensitivity’ and ‘diagnosis’. We searched the key words alongside the following limitations: English language, human studies, and clinical trials. To qualify for inclusion (“inclusion criteria”), studies had to be randomized controlled trials (RCTs). Only articles that were published in English-language peer-reviewed journals were included. Reference lists and reviews were further hand-searched to identify RCTs.

## Results and discussion

A total of 26 studies were found to be relevant for diagnosis and histology, but this number was expanded to include several aspects that are crucial for the narrative section of the review [3–71]. RCTs and clinical trials (CTs) were specifically included. Exclusion criteria included studies that were duplicated, showed overlapping, and, according to the authors, were considered nonrelevant.

Although NCWS is often considered a very recently discovered clinical condition, it was known more than 40 years ago, when Ellis and Linaker presented the case of a 43-year-old patient with diarrhea in the absence of celiac disease, who showed a marked improvement with the removal of gluten from the diet [72]. For forty-two years, the research started to define this entity better, but we are still struggling to arrive at the goal of a clear definition from the clinical, laboratory, and histological point of view. The introduction of the gluten challenge triggering gastrointestinal symptoms following a tightly adherent gluten-free diet (GFD) in individuals without celiac disease reinforced some observations of the 1980s [73]. The idea is that either gluten or wheat may trigger a non-GSE celiac symptomatologic pattern [18, 70]. In this paper, we preferred the use of the label “non-celiac wheat sensitivity” (NCWS), as we consider the real trigger of the symptoms in these patients when they eat wheat to still be undefined. In any case, the label of “non-celiac wheat sensitivity” effectively reflects our poor understanding of this condition and the inability to answer our patients and colleagues correctly [18, 70, 72–76].

In a world dominated by the Internet, it is easy to spread the message that one is better off with a GFD even if one does not have celiac disease or a sensitivity to wheat [77–79]. Thus, a plethora of individual reports and experiences have saturated several channels of information and are showing that ordinary people as well as nonspecialists have found the culprit to be gluten, or preferably wheat, and are choosing a GFD as a lifestyle. Apart from the diabetogenic risk of selecting a GFD if it is not recommended, a GFD has a crucial or nonnegligible effect on one’s financial budget, specifically for people with low incomes [41, 80, 81]. However, there are actual subjects who self-report the onset of gastrointestinal symptoms after consuming some food containing gluten or wheat, and this symptomatology remits after “gluten/wheat” exclusion and recurs following the reintroduction of “gluten/wheat” [19].

Thirty years after its initial description, the diagnosis of NCWS remains sophisticated and vaguely accepted. Currently, to make this diagnosis, we must have a symptomatic reaction to gluten or wheat-containing food in a patient who has been ruled out as having either celiac disease or wheat allergy. In a world where social media is dominating, we often experience individuals who “feel” better applying a GFD to their life either personally or through Internet blogs [77–81].

Gastroenterology, like medicine, is not perfect, and several pathologies are still challenging to correctly define. A similar example is that of irritable bowel syndrome (IBS), which, despite the Roma IV criteria [82], is sometimes difficult to discern from atypical or attenuated forms of inflammatory bowel disease or celiac disease [83].

### Conflicting data from clinical trials

To date, several CTs and RCTs addressing the NCWS issue with different designs, controversial results, and numerous limitations have been published. In Table 1, most of the RCTs on NCWS are listed chronologically with some of the restrictions [14, 61, 84–92]. Conflicting results of clinical trials are a problem but probably not the only one. In examining some of these trials, we highlight these conflicts, but the overemphasis may also be a problem. A randomized, double-blind study reported that dietary gluten, provided in the context of low FODMAPs (fermentable oligosaccharides, disaccharides, monosaccharides, and polyols), induced symptoms compared to placebo in patients who had no histological or serological evidence of celiac disease [93]. The Finnish study performed at the Department of Internal Medicine of the Tampere University Hospital evaluated 93 consecutive adults from health centers spontaneously reporting abdominal symptoms after consumption of cereals. Nine percent of patients had celiac disease, of which 17 had an increased density of gamma delta (γδ) + intraepithelial lymphocytes (IELs) without atrophy.

**Table 1:** Randomized clinical trials

References	Design	N	Dose	Control	Treatment	Wash-out	Run-in	Challenge	Outcome
Biesiekierski [84]	DB-RCT	34	16 g/days	GF bread or muffin	6 weeks	No data	–	Muffins/bread slices	IBS-triggered by gluten challenge
Carroccio [14]	DBPC	50	10 g/days	Xylose	2 weeks	1 weeks	4 weeks elimination	Capsules wheat	GS-Symptoms triggered by wheat
Biesiekierski [85]	DB, cross-over	22	16 g/days	Gluten-low (2 g/days), placebo	2 weeks	2 weeks	6d GFD, 2 weeks low FODMAP	Gluten-rich meals	No symptoms by gluten challenge
Di Sabatino [86]	DBPC, cross-over	61	4.4 g/days		1 weeks	1 weeks	GFD	Capsules gluten	IBS-triggered by gluten challenge
Shahbazkhani [87]	DBPC	72	50 g/days	Sachet powder rice flour	6 weeks	No data	GFD 6 months	Sachet gluten meal	IBS-triggered by gluten challenge
Zanini [88]	DBPC, cross-over	35	10 g/days	Sachet GF-flour	10 days	2 weeks	GFD > 3 months	Sachet gluten	IBS-triggered by gluten challenge
Zanwar [89]	DBPC	60	No data	GF bread	4 weeks	No data	GFD 1 months	Bread	IBS-triggered by gluten challenge
Elli [90]	DBPC, cross-over	98	5.6 g/days	Rice starch	7 days	7 days	GFD × 3 weeks	Capsules gluten	IBS-triggered by gluten challenge
Skodje (2017) [91]	Open challenge	56	120 g/days	No control	3–14 days	No data	GFD 16 months	Bread	GS-Symptoms triggered by wheat
Skodje [61]	DBPC, cross-over	59	5.7 g/days	GF low FODMAP muesli	7 days	7 days	GFD > 6 months	Muesli bars gluten + FOS	IBS-triggered by fructan challenge
Dale [92]	DBPC	20	11 g/days	Rice starch	4 days	3 days	GFD 6 weeks	Muffin gluten	IBS-triggered by gluten challenge

On the other hand, only 8% of patients showed evidence of celiac disease, i.e., both an increase in γδ + IELs and the presence of celiac disease-type HLA. Approximately 20% (one in five) had one or more positive allergy tests for cereals. In the patients who adopted a GFD, all abdominal symptoms were alleviated. However, methodological deficiencies have been delineated [94].

The New Zealand Asthma and Allergy Cohort Study Group reveals how influential the culture in our society is on the choice of a GFD without a proper diagnosis [95]. In only 1% of the 916 enrolled children, physician-diagnosed celiac disease was pronounced. Approximately 5% of these children avoided only gluten.

Interestingly, the analysis of the predictors of gluten avoidance in children without doctor-diagnosed celiac disease suggests critical regional differences in community belief or medical practice considering that the Christchurch census data reveal that 80% are of European ethnicity.

A possible pitfall, in some trials, can be related to the heterogeneity of subjects. In some tests, a mixed population of patients with IBS who likely respond to many dietary exclusion diets, including placebo, has been identified. Methodological issues related to short wash-out periods between challenges contribute to complicating the interpretation of results. Recently, Dale et al. [92] failed to show symptomatic responses to gluten when the challenge was introduced in a blinded fashion. Dale’s study had a rigorous double-blind placebo-controlled design, but FODMAPs were not explicitly removed from the treatment. Dietary management is probably crucial. They supposed that the influence of community belief and culture plays a role that was unknown in the twentieth century or before [96]. On the other hand, to overcome the heterogeneity of the patient population with suspected NCWS included in dietary trials, the two most important studies that concluded that FODMAPs but not gluten cause symptoms [61, 85] planned exclusion criteria, which excluded most of the “real” NCWS patients. To overcome the possible overlap with the initial stage of celiac disease (the so-called “celiac lite”), the authors of both of these studies excluded subjects with IEL count > 25/100 EC (Marsh 1 lesion) who had HLA haplotypes predisposing to celiac disease (DQ2 and DQ8) and some other pathological conditions mimicking the initial morphological phase of GSE. Although these rigorous exclusion criteria must be considered excellent with respect to the “celiac lite” problem, it must be emphasized that the majority of the subjects suffering from NCWS had an intraepithelial infiltration of the duodenal mucosa; furthermore, approximately 50% of the NCWS subjects display DQ2 or DQ8 haplotypes, a percentage that is higher than that reported in the general population. Thus, the exclusion criteria of those studies excluded the “inflamed patients”, i.e., those who were more likely to suffer from a real NCWS. Regarding mucosal inflammation in patients with NCWS, recent studies based on confocal endoscopy have demonstrated in a large percentage of subjects with IBS that wheat administration on the mucosa determined the break of the tight junctions and lymphocytic infiltration. Numerous patients with IBS have atypical food allergies, which are not associated with immunoglobulin E [97]. Thus, once again, there is evidence that duodenal mucosal inflammation, although in the absence of villi atrophy, characterizes NCWS.

Moreover, the two most common GSE mimickers, excluding inflammatory bowel disease, are gastric Helicobacter pylori (H. pylori) infection and medications, especially nonsteroidal anti-inflammatory drugs (NSAIDs) or proton-pump inhibitors (PPIs) and an increased IEL count is observed in the duodenum of patients with H. pylori gastritis [98].

Since the first report of NCWS in 1978 [72], the approach to this entity has been troubled by scientific and nonscientific reports revealing considerable debate as well as a conspicuous conflict of interest. The terminology “gluten sensitivity” (GS) infers an immunologic reactivity to “gluten”. Thus, gluten sensitivity that may be triggered or not triggered by gluten includes celiac disease. It indeed enhances confusion. Gastrointestinal and nutritionist professional meetings have discussed this condition since early 2000 [19, 99, 100]. In the 2012 report assembling scientists and physicians from several countries and issued by the Center for Celiac Research, located at the University of Maryland School of Medicine, Baltimore, United States, Sapone et al. [99] defined NCWS (or GS) as a reaction to gluten in which both allergic and autoimmune mechanisms have been accurately excluded. The report emphasizes the diagnosis of exclusion, but one of the major problems in celiac research is the accuracy of the diagnosis. Of course, there are clear-cut cases of celiac disease, but a few instances of IELs may easily cross the borders of the categories. Numerous gastrointestinal pathologists know the “difficult” cases that need to be discussed at the multidisciplinary team meetings. In a Canadian report, Armstrong et al. [101] indicated that subjects harboring NCWS should have typical duodenal histopathology and negative specific celiac serology, but some nonspecific biomarkers such as antigliadin antibodies may be positive biochemically, but the implementation of a GFD helping to resolve the symptomatology obscures the boundaries with patients with celiac disease.

### Current criteria for NCWS diagnosis

To make the NCWS diagnosis, we must have a symptomatic reaction to wheat-containing food in a patient who has been ruled out as having either celiac disease or wheat allergy. Catassi et al. proposed a 2-step algorithm at the Salerno conference [18, 20]. Once a symptom-accompanying patient presents to the physician, celiac disease and wheat allergy need to be ruled out first, and a GFD is suggested for six weeks. Skin prick testing, serum IgE testing and oral food challenges remain the current gold standard tests for anaphylactic (immediate) type food allergies, while serum IgG testing, electrodermal testing, cytotoxic testing, provocation/neutralization, and applied kinesiology are unproven unaccepted methods to diagnose it [102]. The symptoms of the patient are scored according to the global symptoms rating scale before and following the six weeks of GFD. If the patient has a resolution of the symptoms after the GFD, the diagnosis of NCWS is made; otherwise, the patient requires additional investigations. After this 1st step, the 2nd step encompasses a double-blind placebo-controlled challenge with a crossover design. This study design is needed for the confirmation of the diagnosis of NWGS in patients responding to treatment with a GFD [20, 42]. According to the Salerno criteria [20], there is a 1-week challenge, which involves the administration of capsules containing 8 g of gluten with at least 0.3 g of amylase trypsin inhibitors (ATIs) embedded in a FODMAP-free vehicle and a gluten-free placebo. The challenge should follow a 1-week wash-out. It seems that a subtype may be delineated, incorporating patients with IBS-type symptoms and labeled “gluten or wheat-sensitive IBS” [18].

### Is there a role for mucosal histology in the NCWS diagnosis?

The histopathology of NCWS is even still, like the clinical and laboratory features, a point of debate, and in many cases, there is extreme confusion. The fundamental assumption for a correct histopathological evaluation is the clinical and methodological assumption, namely, a precise selection of patients based on clinical and laboratory characteristics [20]. Furthermore, an exact number of correctly oriented biopsies (greater than or equal to 4) as in the case of celiac disease in the second duodenal portion and the bulb need to be carried out. The orientation of the same biopsies in which the use of cellulose acetate filters that are already cut for this purpose is also recommended to avoid false atrophies and imprecise T lymphocyte counts, and the role of some infections, such as in *Helicobacter pylori*-related gastritis or infestations caused by *Giardia Lamblia* or *Cryptosporidium*, which can cause an increased number of IELs (intraepithelial lymphocytosis, IEL) without architectural abnormalities, cannot be sufficiently emphasized [98, 103–109]. Moreover, several drugs and autoimmune disorders produce the same histology findings. Other reported conditions associated with an increased number of IELs include Hashimoto thyroiditis, Graves’ disease, rheumatoid arthritis, psoriasis, multiple sclerosis, systemic lupus erythematosus and common variable immune deficiency.

Most of the work on the topic of NCWS considers the duodenal mucosa healthy or almost healthy, and without indicating morphological aspects that are useful when suspecting the condition, it can be considered useless. The only experience, to our knowledge, that may suggest a morphological framework was the cooperative work of the Brescia and Palermo groups [110–112] and the crucial Brescia experience in which, based on careful clinical and laboratory selection based on the Salerno criteria [20], biopsies were performed in 18 patients and 10 controls.

**Fig. 1:**
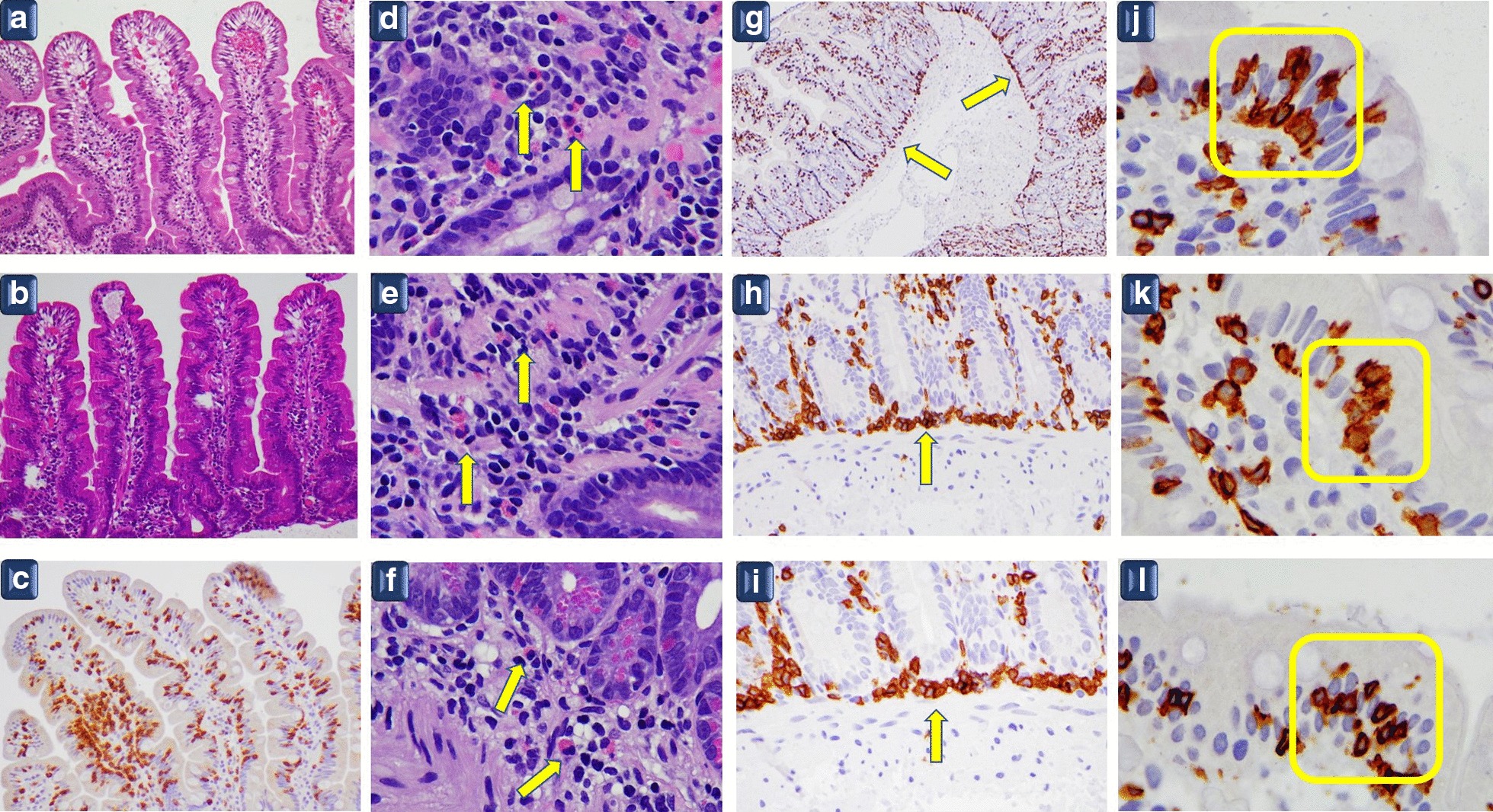
**a**–**c** Normal villi with T lymphocytes in the normal range (< 25 for 100 epithelial cells). **a**–**b** Hematoxylin and eosin staining × 10; **c** CD3 immunostaining × 10 original magnification. **d**–**f**: Eosinophils in the lamina propria (arrows). Hematoxylin and eosin staining × 100 original magnification. **g**–**i** Linear distribution of T lymphocytes in the deeper part of the mucosa over the muscularis mucosae, the outermost layer of the mucosa (arrows). CD3 immunostaining **g** × 10 original magnification **h**–**i** × 40 original magnification. **j**–**l** Cluster of 5–6 T lymphocytes in the superficial epithelium of villi (yellow rectangles) CD3 immunostaining × 100 original magnification

The morphological elements evidenced in the Brescia experience are as follows:

A A “nearly” standard number of T lymphocytes (< 25 for 100 epithelial cells)
B A peculiar disposition of T lymphocytes in a small “cluster” of 4 or 5 cells in the superficial epithelium
C The linear distribution of T lymphocytes in the deeper part of the lamina propria of the mucosa over the muscularis mucosae (*lamina muscularis mucosae*)
D An increased number of eosinophils in the lamina propria (> 5 cells per high-power field, HPF) **(**Fig. 1).

The presence of eosinophils may suggest a condition similar to food allergies. Further prospective studies are needed to confirm the Brescia-Palermo findings and their specificity. Although the term “nearly” may not satisfy physicians, the heterogeneity of studies and data have indicated that at least some of the subjects suffering from NCWS may have increased IELs [39, 83, 113]. Another point that deserves attention in future studies is the “site of intestinal inflammation” in NCWS. Almost all the reviews, to date, have focused on the duodenal mucosa, probably due to the need to exclude celiac disease in a patient who reports symptoms induced by wheat ingestion. However, it must be remembered that most patients with NCWS have an IBS-like clinical presentation, suggesting “clinical involvement” of the colon. According to the clinical presentation, a recent study of the Palermo and Brescia groups showed a relevant eosinophilic infiltration in the rectal mucosa of patients with NCWS, which was more intense in the rectal than in the duodenal mucosa. Interestingly, the numbers of eosinophils in the duodenal mucosa were higher in patients with NCWS with dyspepsia than in patients with NCWS without upper digestive tract symptoms [112]. Overall, these data could indicate that NCWS is an inflammatory condition that involves the entire gastrointestinal tract and that the site of dominant inflammation drives specific clinical symptoms.

### Diagnostic challenges

As pediatricians, gastroenterologists, and pathologists, it is mandatory to exclude other diseases, such as autoimmune conditions, lactose and fructose intolerance, inflammatory bowel disease (ulcerative colitis, Crohn’s disease, indeterminate colitis), and pancreatic insufficiency before the diagnosis of NCWS is proposed [20]. The likeness of the clinical presentation of celiac disease and NCWS may be striking. Although some may consider NCWS and celiac disease two “sisters” with similar features, there are substantial differences between these two conditions, and probably, only a minor percentage of patients with NCWS suffer from a condition near celiac disease; these probably include the NCWS cases among first-degree relatives of patients with celiac disease. Furthermore, in some cases, discernment from psychosomatic symptoms may be impossible. Apart from lethargy, extraintestinal symptoms may include fatigue, skin rash, gynecologic troubles (recurrent vaginitis and cystitis in the potential setting of endometriosis), headache, joint and muscle pain (fibromyalgia or fibromyalgia-like syndrome), leg or arm numbness, and anemia as well as neurologic disorders such as depression, anxiety, and psychosis. It seems that a GFD is particularly successful in improving these extraintestinal manifestations [42, 102, 112].

### Pathogenetic hypotheses

The lack of clarity in the definition, as well as the presence of controversies in clinical trials, suggest that the etiopathogenesis remains poorly understood. Alterations in intestinal permeability, as well as abnormal motility and stimulation of the gut, have been postulated. Gliadin can trigger hypercontractility of smooth muscle and cholinergic nerve dysfunction in some animal models (e.g., mice) expressing the human leukocyte antigen (HLA)-DQ8 genes without causing duodenal atrophy of the duodenal mucosa [101, 114]. In particular, gut dysfunction has been demonstrated in gluten-sensitive HLA-DQ8 transgenic mice. In a study, acetylcholine release, small intestinal contractility, and epithelial ion transport were measured in HLA-DQ8 mice sensitized and gavaged with gliadin three times per week for three weeks [115]. Increased acetylcholine release from the myenteric plexus, muscle hypercontractility, and increased active ion transport were demonstrated in sensitized mice. Changes in muscle contractility were normalized in DQ8 mice after gluten withdrawal. Recruitment of IELs, macrophages, and FOX-P3 was observed in sensitized mice in the absence of intestinal atrophy. HLA-DQ6 controls did not exhibit the abnormalities in gut function observed in DQ8 mice. Overall, these experimental data provide evidence that “gluten sensitivity” in HLA-DQ8 mice may lead to altered water movements and dysmotility. Some divergences in intestinal permeability in NCWS have also been demonstrated in celiac disease [116, 117].

Concerning the potential trigger protein and nonprotein fractions in the wheat kernel, gluten constitutes the most abundant protein fraction in wheat (~ 80%). In comparison, ATIs account for approximately 3% [118], and other proteins (e.g., agglutinin) in wheat germ account for the remainder. As quoted above, there is no proof that gluten is the real culprit in NCWS. On the other hand, ATIs could be an “interesting” pathogenetic candidate. Although different polymorphisms of Toll-like receptor 4 (TLR4) have not been found in celiac disease [119], it seems that the TLR4-MD2-CD14 complex may be a potent activator of innate immune responses in monocytes, macrophages, and dendritic cells through Toll-like receptors and may play a role in NCWS [60, 120, 121]. MD2 is a secreted protein that interacts with TLR4, binds the lipid A moiety of lipopolysaccharides, and facilitates the creation of TLR4 dimers that are intimately required for downstream signaling and activation of the NF-κB (nuclear factor kappa light chain enhancer of activated B cells) pathway, while CD14 is a glycosyl-phosphatidylinositol (GPI)-linked protein found on the cell membrane in lipid rafts (microdomains at the plasmatic membrane with definite lipid and protein composition) and functions to transfer lipopolysaccharides from the bacterial cell wall to the MD2/TLR4 complex [122].

Furthermore, wheat is a rich source of the fructose molecule polymer fructooligosaccharides (FOS), and this has been suggested to trigger symptoms in some patients with IBS symptomatology. However, it must be stressed that the malabsorption of short-chain carbohydrates can determine intestinal symptoms, whereas it is challenging to link this mechanism with the frequent extraintestinal symptoms reported by patients suffering from NCWS.

### Social and media aspects in choosing a GFD

It is entirely premature to consider the rate of this condition because the criteria used for diagnosing it are still at the initial stages of the process (13). There are incredible difficulties in assessing the prevalence of NCWS correctly because most of the patients are diagnosed by practitioners or by the patients themselves. After all, the information is overwhelming on social media (see below). A New Zealand study highlights some concerning data showing that despite only 1% of children having celiac disease, 5% are on a GFD [95]. In the United States, a 0.55% prevalence rate of subjects on a self-reported GFD of 7762 persons (unselected) aged six years or older between 2009 and 2010 was found [123]. This rate is the lowest prevalence of self-reported NCWS in the literature, and it must be emphasized that this study was performed before the current interest and knowledge about NCWS. On the other hand, NCWS may affect up to 6% of the U.S. population [123] and worldwide up to 13% if self-reported NCWS is considered [124, 125]. On the other hand, these data may not be reliable because of the subjectivity of some symptoms, the nonmedical approach, and the lack of a trustworthy biomarker estimating an exact prevalence rate [126].

In both Europe and North America, adopting a GFD has become increasingly popular, and interest in a GFD has been growing from 2004 to 2018 (https://trends.google.com/trends), eclipsing the low-carbohydrate diet and fat-free diets. The so-called “clean diets” are bombarding social media and news outlets, and the GFD market is incredibly lucrative. Adopting a GFD and not having NCWS may be disastrous for the organism if a correct balance of proteins, carbohydrates, and lipids is not applied. Selecting a GFD and having NCWS may be the only choice if other conditions have been adequately ruled out by an expert healthcare practitioner. Because derivatives of gluten-rich grains are relevant sources of nutrients in the general diet, their exclusion from the diet could potentially have significant effects on nutritional status. For example, in patients with NCWS on a wheat-free diet, a higher frequency of osteopenia and osteoporosis than in controls has been reported [127]. In addition to the likelihood of nutritional deficiencies following a nonsupplemented and unsupervised GFD, this dietary restriction has been shown to disturb the richness and composition of gut microbiota. At the end of the 2nd decade of this 3rd millennium, the number of individuals embracing a GFD appears to be much higher than the projected number of patients with celiac disease, and the growth has been exponential for several countries. Much of the world’s manufacture of wheat is consumed after it has been processed into bread, several baked goods, noodles, and pasta, while in the Middle East and North Africa, bulgur and couscous are the main products. The choice of GFD fueled a global market of gluten-free products approaching $2.5 billion (United States) in global sales in 2010. Nevertheless, this trend is not going to stop because of social media and scientific or pseudoscientific reports of gluten being the culprit for both intestinal and extraintestinal diseases, such as attention deficit hyperactivity disorder, autism, mood disorders and psychosis [17, 19, 41, 128–134]. This trend is reinforced by the belief that, along with celiac disease, other conditions related to the ingestion of gluten have emerged. The Mediterranean diet implies an abundance of plant foods, including fruits, vegetables, whole grains, nuts, and legumes, which are minimally processed, seasonally fresh, and grown locally. Olive oil, as the principal source of fat, cheese, and yogurt, is consumed daily in low to moderate amounts. Although the Mediterranean diet is probably now the best diet to avoid cardiovascular diseases as well as rheumatic and oncological diseases, GFD is embraced with the spirit of madness, evoking problems related to gluten that have no substantial background [135–139]. Doubtless, the introduction of gluten-containing grains, which occurred approximately 10,000 years ago with the advent of agriculture, embodies an evolutionary challenge of human beings. It obviously created the conditions for human diseases related to gluten exposure to be presented. Although cofactors in triggering these diseases are largely unknown, the balance of a diet for children, youth, and adults in the 3rd millennium is full of pitfalls because of food processing and industry interests. The importance of excluding wheat from the Mediterranean diet in patients without celiac disease may have consequences for the gut microbiome, as evidenced in some studies of patients with inflammatory bowel disease using a different diet [137]. At first glance, the South Asian diet may be healthier because of the lack of bloating complaints [140, 141]. However, the diffusion of noodles in North China substantially compares to the use of wheat in the Western region. Table 2 shows the advantages and disadvantages of choosing a GFD for a patient not suffering from “typical” wheat-related diseases. This table is far from perfect but collects the experience of the authors and data from the literature. Substantially, the downgrading of the difficulties in this table is mostly influenced by community factors, which are primarily associated with social media influences. The persuasive power of social media may skew the decision of the single individual in lifestyle choices [81, 142–146]. The influence on social behavior can be challenging with social impairment; these individuals can experience restriction and even deprivation. This challenge as well as the ignorance of medical research may shape the appropriateness of choices of future generations.

**Table 2:** Conditions that may improve or worsen in non-celiac symptomatic subjects by adopting a gluten-free diet

Improve	Worsen
Atopy	Coronary artery disease
Attention deficit-hyperactivity disorders	Fiber deficiency
Autism spectrum disorder	Financial burden
Endometriosis	Hyperglycemia
Fibromyalgia	Hyperlipidemia
Irritable bowel syndrome symptoms	Micronutrient deficiency
Obesity/overweight	Social deprivation
Psychosis and schizophrenia	Social impairment
Social inclusion	Social restriction

## Conclusions

The number of reports on GFD and the harmful effects of gluten has been increasing tremendously in social media and scientific journals over the past ten years. Although the Salerno criteria helped to delineate the NCWS group better, there is still much work to do in this field. In this review, the clinical and histological elements evidenced in the experience of two major Italian centers (Palermo and Brescia) and one Canadian center (Edmonton) have been characterized, but we must affirm that no picture can be considered pathognomonic. Unfortunately, we still have an inaccurate histologic marker for NCWS diagnosis, and duodenal biopsy is undoubtedly useful to rule out a seronegative celiac disease but not for a “positive diagnosis” of NCWS. From a clinical point of view, we agree that NCWS is not the only cause of IBS, which is a complex, multifactorial condition. Wheat contains many potentially immunogenic proteins in addition to gluten, and nonprotein fractions could trigger symptoms in patients with functional disorders. Beyond functional disorders, future studies will define whether any wheat component can be implied in the pathogenesis of inflammation and autoimmune diseases.

Similarly, studies investigating biomarkers and epigenetics are needed to identify the subpopulations that will benefit most from a GFD. From a social point of view, although most individuals consuming a wheat-containing diet do not have adverse reactions, social influence on nutrition has reshaped some aspects of the dietary habits of our society and probably forced them to consume a wheat-free diet.

## Data Availability

All data and PDFs have been deposited at the Department of Laboratory Medicine and Pathology, University of Alberta, Canada. All data and PDFs will be deposited with the current Anatomic Pathology Site Chief (Dr. Judith Hugh), University of Alberta Hospital, 8440 112 St, T6G 2B7 Edmonton, AB, Canada (Judith.Hugh@albertaprecisionlabs.ca). Alternatively, all authors will have the files. Each author will be available to provide them following a reasonable request. The original slides of the histopathology from patients affected with non-celiac wheat sensitivity are located at the Institute of Pathology ASST-*Spedali Civili*, Brescia, Italy. Dr. V. Villanacci will be responsible to provide recuts from the paraffin blocks following a reasonable request.

## Abbreviations

ATIs: Amylase trypsin inhibitors
CTs: Clinical trials
DB: Double-blind
DBPC: Double-blind placebo-controlled
FODMAPs: Fermentable oligosaccharides, disaccharides, monosaccharides, and polyols
FOS: Fructooligosaccharides
GFD: Gluten-free diet
GS: Gluten sensitivity
GPI: Glycosyl-phosphatidylinositol
H. pylori: Helicobacter pylori
HLA: Human leukocyte antigen
IBS: Inflammatory bowel syndrome
IEL: Intraepithelial lymphocytosis
IELs: Intraepithelial lypmphocytes
Mos: Months
NF-κB: Nuclear factor kappa light chain enhancer of activated B cells
NCWS: Non-celiac gluten or wheat sensitivity
NSAIDs: Nonsteroidal anti-inflammatory drugs
PPIs: Proton-pump inhibitors
RCTs: Randomized controlled trials
TLR4: Toll-like receptor 4
wks: Weeks

## References

[1] Fasano A (2003). Celiac disease–how to handle a clinical chameleon. N Engl J Med.

[2] Fasano A, Sapone A, Zevallos V, Schuppan D (2015). Nonceliac gluten sensitivity. Gastroenterology.

[3] Akhondi H, Ross AB. Gluten and associated medical problems. In: StatPearls*.* edn. Treasure Island (FL); 2019. 30860740

[4] Barbaro MR, Cremon C, Stanghellini V, Barbara G. Recent advances in understanding non-celiac gluten sensitivity. F1000Res 2018, 7. 10.12688/f1000research.15849.1 PMC6182669 30363819

[5] Bardella MT, Elli L, Ferretti F (2016). Non celiac gluten sensitivity. Curr Gastroenterol Rep.

[6] Bathrellou E, Kontogianni MD, Panagiotakos DB (2018). Celiac disease and non-celiac gluten or wheat sensitivity and health in later life: a review. Maturitas.

[7] Biesiekierski JR, Muir JG, Gibson PR (2013). Is gluten a cause of gastrointestinal symptoms in people without celiac disease?. Curr Allergy Asthma Rep.

[8] Bonciolini V, Bianchi B, Del Bianco E, Verdelli A, Caproni M (2015). Cutaneous manifestations of non-celiac gluten sensitivity: clinical histological and immunopathological features. Nutrients.

[9] Brusca I (2015). Overview of biomarkers for diagnosis and monitoring of celiac disease. Adv Clin Chem.

[10] Cabrera-Chavez F, Dezar GV, Islas-Zamorano AP, Espinoza-Alderete JG, Vergara-Jimenez MJ, Magana-Ordorica D, Ontiveros N (2017). Prevalence of self-reported gluten sensitivity and adherence to a gluten-free diet in Argentinian adult population. Nutrients.

[11] Capannolo A, Viscido A, Barkad MA, Valerii G, Ciccone F, Melideo D, Frieri G, Latella G (2015). Non-celiac gluten sensitivity among patients perceiving gluten-related symptoms. Digestion.

[12] Carroccio A, Giambalvo O, Blasca F, Iacobucci R, D'Alcamo A, Mansueto P (2017). Self-reported non-celiac wheat sensitivity in high school students: demographic and clinical characteristics. Nutrients.

[13] Carroccio A, Mansueto P, D'Alcamo A, Iacono G (2013). Non-celiac wheat sensitivity as an allergic condition: personal experience and narrative review. Am J Gastroenterol.

[14] Carroccio A, Mansueto P, Iacono G, Soresi M, D'Alcamo A, Cavataio F, Brusca I, Florena AM, Ambrosiano G, Seidita A (2012). Non-celiac wheat sensitivity diagnosed by double-blind placebo-controlled challenge: exploring a new clinical entity. Am J Gastroenterol.

[15] Casella G, Di Bella C, Salemme M, Villanacci V, Antonelli E, Baldini V, Bassotti G (2015). Celiac disease, non-celiac gluten sensitivity and inflammatory bowel disease. Minerva Gastroenterol Dietol.

[16] Casella G, Villanacci V, Di Bella C, Bassotti G, Bold J, Rostami K (2018). Non celiac gluten sensitivity and diagnostic challenges. Gastroenterol Hepatol Bed Bench.

[17] Catassi C (2015). Gluten sensitivity. Ann Nutr Metab.

[18] Catassi C, Alaedini A, Bojarski C, Bonaz B, Bouma G, Carroccio A, Castillejo G, De Magistris L, Dieterich W, Di Liberto D (2017). The Overlapping area of non-celiac gluten sensitivity (NCGS) and wheat-sensitive irritable bowel syndrome (IBS): an update. Nutrients.

[19] Catassi C, Bai JC, Bonaz B, Bouma G, Calabro A, Carroccio A, Castillejo G, Ciacci C, Cristofori F, Dolinsek J (2013). Non-Celiac Gluten sensitivity: the new frontier of gluten related disorders. Nutrients.

[20] Catassi C, Elli L, Bonaz B, Bouma G, Carroccio A, Castillejo G, Cellier C, Cristofori F, de Magistris L, Dolinsek J (2015). Diagnosis of non-celiac gluten sensitivity (NCGS): the Salerno experts' criteria. Nutrients.

[21] Czaja-Bulsa G (2015). Non coeliac gluten sensitivity—a new disease with gluten intolerance. Clin Nutr.

[22] D'Alcamo A, Mansueto P, Soresi M, Iacobucci R, Blasca F, Geraci G, Cavataio F, Fayer F, Arini A, Di Stefano L (2017). Contact dermatitis due to nickel allergy in patients suffering from non-celiac wheat sensitivity. Nutrients.

[23] DeGeeter C, Guandalini S (2018). Food sensitivities: fact versus fiction. Gastroenterol Clin North Am.

[24] Di Stefano M, Pesatori EV, Manfredi GF, De Amici M, Grandi G, Gabriele A, Iozzi D, Di Fede G (2018). Non-celiac gluten sensitivity in patients with severe abdominal pain and bloating: the accuracy of ALCAT 5. Clin Nutr ESPEN.

[25] Elli L, Branchi F, Tomba C, Villalta D, Norsa L, Ferretti F, Roncoroni L, Bardella MT (2015). Diagnosis of gluten related disorders: Celiac disease, wheat allergy and non-celiac gluten sensitivity. World J Gastroenterol.

[26] Elli L, Marinoni B (2019). Gluten rhapsody. Nutrients.

[27] Elli L, Roncoroni L, Bardella MT (2015). Non-celiac gluten sensitivity: time for sifting the grain. World J Gastroenterol.

[28] Elli L, Villalta D, Roncoroni L, Barisani D, Ferrero S, Pellegrini N, Bardella MT, Valiante F, Tomba C, Carroccio A (2017). Nomenclature and diagnosis of gluten-related disorders: a position statement by the Italian Association of Hospital Gastroenterologists and Endoscopists (AIGO). Dig Liver Dis.

[29] Figueroa-Salcido OG, Ontiveros N, Cabrera-Chavez F (2019). Gluten vehicle and placebo for non-celiac gluten sensitivity assessment. Medicina (Kaunas).

[30] Francavilla R, Cristofori F, Verzillo L, Gentile A, Castellaneta S, Polloni C, Giorgio V, Verduci E, D'Angelo E, Dellatte S (2018). Randomized double-blind placebo-controlled crossover trial for the diagnosis of non-celiac gluten sensitivity in children. Am J Gastroenterol.

[31] Harper L, Bold J (2018). An exploration into the motivation for gluten avoidance in the absence of coeliac disease. Gastroenterol Hepatol Bed Bench.

[32] Heydari F, Rostami-Nejad M, Moheb-Alian A, Mollahoseini MH, Rostami K, Pourhoseingholi MA, Aghamohammadi E, Zali MR (2018). Serum cytokines profile in treated celiac disease compared with non-celiac gluten sensitivity and control: a marker for differentiation. J Gastrointestin Liver Dis.

[33] Hoffmanova I (2019). Non-celiac gluten/wheat sensitivity: still more questions than answers. Vnitr Lek.

[34] Ierardi E, Losurdo G, Iannone A, Piscitelli D, Amoruso A, Barone M, Principi M, Pisani A, Di Leo A (2017). Lymphocytic duodenitis or microscopic enteritis and gluten-related conditions: what needs to be explored?. Ann Gastroenterol.

[35] Igbinedion SO, Ansari J, Vasikaran A, Gavins FN, Jordan P, Boktor M, Alexander JS (2017). Non-celiac gluten sensitivity: all wheat attack is not celiac. World J Gastroenterol.

[36] Infantino M, Manfredi M, Meacci F, Grossi V, Severino M, Benucci M, Bellio E, Bellio V, Nucci A, Zolfanelli F (2015). Diagnostic accuracy of anti-gliadin antibodies in Non Celiac Gluten Sensitivity (NCGS) patients: a dual statistical approach. Clin Chim Acta.

[37] Isasi C, Tejerina E, Moran LM (2016). Non-celiac gluten sensitivity and rheumatic diseases. Reumatol Clin.

[38] Khan A, Suarez MG, Murray JA (2020). Nonceliac gluten and wheat sensitivity. Clin Gastroenterol Hepatol.

[39] Kirmizi A, Kalkan C, Yuksel S, Gencturk Z, Savas B, Soykan I, Cetinkaya H, Ensari A (2018). Discriminant value of IEL counts and distribution pattern through the spectrum of gluten sensitivity: a simple diagnostic approach. Virchows Arch.

[40] Levy J, Bernstein L, Silber N (2014). Celiac disease: an immune dysregulation syndrome. Curr Probl Pediatr Adolesc Health Care.

[41] Lionetti E, Leonardi S, Franzonello C, Mancardi M, Ruggieri M, Catassi C (2015). Gluten psychosis: confirmation of a new clinical entity. Nutrients.

[42] Lionetti E, Pulvirenti A, Vallorani M, Catassi G, Verma AK, Gatti S, Catassi C (2017). Re-challenge studies in non-celiac gluten sensitivity: a systematic review and meta-analysis. Front Physiol.

[43] Livzan MA, Osipenko MF, Zayakina NV, Krolevets TS (2016). Principles of diagnosis of gluten-associated diseases. Eksp Klin Gastroenterol.

[44] Llanos-Chea A, Fasano A (2018). Gluten and functional abdominal pain disorders in children. Nutrients.

[45] Lu Z, Zhang H, Luoto S, Ren X (2018). Gluten-free living in China: the characteristics, food choices and difficulties in following a gluten-free diet—an online survey. Appetite.

[46] Lundin KE, Alaedini A (2012). Non-celiac gluten sensitivity. Gastrointest Endosc Clin N Am.

[47] Makharia A, Catassi C, Makharia GK (2015). The overlap between irritable bowel syndrome and non-celiac gluten sensitivity: a clinical dilemma. Nutrients.

[48] Mansueto P, D'Alcamo A, Seidita A, Carroccio A (2015). Food allergy in irritable bowel syndrome: the case of non-celiac wheat sensitivity. World J Gastroenterol.

[49] Mansueto P, Seidita A, D'Alcamo A, Carroccio A (2014). Non-celiac gluten sensitivity: literature review. J Am Coll Nutr.

[50] Mansueto P, Soresi M, La Blasca F, Fayer F, D'Alcamo A, Carroccio A (2019). Body mass index and associated clinical variables in patients with non-celiac wheat sensitivity. Nutrients.

[51] Marchioni Beery RM, Birk JW (2015). Wheat-related disorders reviewed: making a grain of sense. Expert Rev Gastroenterol Hepatol.

[52] McAllister BP, Williams E, Clarke K (2018). A comprehensive review of celiac disease/gluten-sensitive enteropathies. Clin Rev Allergy Immunol.

[53] Nijeboer P, Bontkes HJ, Mulder CJ, Bouma G (2013). Non-celiac gluten sensitivity. Is it in the gluten or the grain?. J Gastrointestin Liver Dis.

[54] Rathi PM, Zanwar VG (2016). Non-celiac gluten sensitivity (NCGS). J Assoc Phys. India.

[55] Reese I, Schafer C, Kleine-Tebbe J, Ahrens B, Bachmann O, Ballmer-Weber B, Beyer K, Bischoff SC, Blumchen K, Dolle S (2018). Non-celiac gluten/wheat sensitivity (NCGS)-a currently undefined disorder without validated diagnostic criteria and of unknown prevalence: Position statement of the task force on food allergy of the German Society of Allergology and Clinical Immunology (DGAKI). Allergo J Int.

[56] Rotondi Aufiero V, Fasano A, Mazzarella G (2018). Non-celiac gluten sensitivity: how its gut immune activation and potential dietary management differ from celiac disease. Mol Nutr Food Res.

[57] Rua EC, Drut R, Pena AS (2013). Non-celiac wheat sensitivity is not a new entity. Am J Gastroenterol.

[58] Ruemmele FM (2018). Non-celiac gluten sensitivity: a challenging diagnosis in children with abdominal pain. Ann Nutr Metab.

[59] Sanders DS, Aziz I (2012). Non-celiac wheat sensitivity: separating the wheat from the chat!. Am J Gastroenterol.

[60] Schuppan D, Pickert G, Ashfaq-Khan M, Zevallos V (2015). Non-celiac wheat sensitivity: differential diagnosis, triggers and implications. Best Pract Res Clin Gastroenterol.

[61] Skodje GI, Sarna VK, Minelle IH, Rolfsen KL, Muir JG, Gibson PR, Veierod MB, Henriksen C, Lundin KEA (2018). Fructan, rather than gluten, induces symptoms in patients with self-reported non-celiac gluten sensitivity. Gastroenterology.

[62] Soares RLS (2018). Irritable bowel syndrome, food intolerance and non-celiac gluten sensitivity. A new clinical challenge. Arq Gastroenterol.

[63] Tanveer M, Ahmed A (2019). Non-celiac gluten sensitivity: a systematic review. J Coll Physicians Surg Pak.

[64] Tovoli F, Granito A, Negrini G, Guidetti E, Faggiano C, Bolondi L (2019). Long term effects of gluten-free diet in non-celiac wheat sensitivity. Clin Nutr.

[65] Valenti S, Corica D, Ricciardi L, Romano C (2017). Gluten-related disorders: certainties, questions and doubts. Ann Med.

[66] Vasagar B, Cox J, Herion JT, Ivanoff E (2017). World epidemiology of non-celiac gluten sensitivity. Minerva Gastroenterol Dietol.

[67] Volta U, Caio G, De Giorgio R, Henriksen C, Skodje G, Lundin KE (2015). Non-celiac gluten sensitivity: a work-in-progress entity in the spectrum of wheat-related disorders. Best Pract Res Clin Gastroenterol.

[68] Volta U, Caio G, Tovoli F, De Giorgio R (2013). Non-celiac gluten sensitivity: questions still to be answered despite increasing awareness. Cell Mol Immunol.

[69] Volta U, De Giorgio R, Caio G, Uhde M, Manfredini R, Alaedini A (2019). Nonceliac wheat sensitivity: an immune-mediated condition with systemic manifestations. Gastroenterol Clin North Am.

[70] Volta U, Pinto-Sanchez MI, Boschetti E, Caio G, De Giorgio R, Verdu EF (2016). Dietary triggers in irritable bowel syndrome: is there a role for gluten?. J Neurogastroenterol Motil.

[71] Wieser H, Scherf KA (2018). Preparation of a defined gluten hydrolysate for diagnosis and clinical investigations of wheat hypersensitivities. Nutrients.

[72] Ellis A, Linaker BD (1978). Non-coeliac gluten sensitivity?. Lancet.

[73] Cooper BT, Holmes GK, Ferguson R, Thompson RA, Allan RN, Cooke WT (1980). Gluten-sensitive diarrhea without evidence of celiac disease. Gastroenterology.

[74] Ngatchu T, Cash D, Langman G (2010). Is indeterminate colitis really indeterminate?. Gut.

[75] Tremaine WJ (2012). Is indeterminate colitis determinable?. Curr Gastroenterol Rep.

[76] Odze RD (2015). A contemporary and critical appraisal of 'indeterminate colitis'. Mod Pathol.

[77] Park K, Harris M, Khavari N, Khosla C. Rationale for using social media to collect patient-reported outcomes in patients with celiac disease. J Gastrointest Dig Syst 2014, 4(1). 10.4172/2161-069X.1000166 PMC4226063 25392743

[78] Kiedrowski M, Mroz A, Gajewska D, Nurzynski P, Deptala A (2017). Celiac disease on YouTube—A study of the Polish content available on the popular video-sharing website. Pol Merkur Lekarski.

[79] Haas K, Martin A, Park KT (2017). Text message intervention (TEACH) improves quality of life and patient activation in celiac disease: a randomized clinical trial. J Pediatr.

[80] Niland B, Cash BD (2018). Health benefits and adverse effects of a gluten-free diet in non-celiac disease patients. Gastroenterol Hepatol (N Y).

[81] Paganizza S, Zanotti R, D'Odorico A, Scapolo P, Canova C (2019). Is Adherence to a gluten-free diet by adult patients with celiac disease influenced by their knowledge of the gluten content of foods?. Gastroenterol Nurs.

[82] Drossman DA (2016). Functional gastrointestinal disorders: history, pathophysiology, clinical features and Rome IV. Gastroenterology.

[83] Volta U, Tovoli F, Cicola R, Parisi C, Fabbri A, Piscaglia M, Fiorini E, Caio G (2012). Serological tests in gluten sensitivity (nonceliac gluten intolerance). J Clin Gastroenterol.

[84] Biesiekierski JR, Newnham ED, Irving PM, Barrett JS, Haines M, Doecke JD, Shepherd SJ, Muir JG, Gibson PR (2011). Gluten causes gastrointestinal symptoms in subjects without celiac disease: a double-blind randomized placebo-controlled trial. Am J Gastroenterol.

[85] Biesiekierski JR, Peters SL, Newnham ED, Rosella O, Muir JG, Gibson PR (2013). No effects of gluten in patients with self-reported non-celiac gluten sensitivity after dietary reduction of fermentable, poorly absorbed, short-chain carbohydrates. Gastroenterology.

[86] Di Sabatino A, Volta U, Salvatore C, Biancheri P, Caio G, De Giorgio R, Di Stefano M, Corazza GR (2015). Small amounts of gluten in subjects with suspected nonceliac gluten sensitivity: a randomized, double-blind, placebo-controlled, cross-over trial. Clin Gastroenterol Hepatol.

[87] Shahbazkhani B, Sadeghi A, Malekzadeh R, Khatavi F, Etemadi M, Kalantri E, Rostami-Nejad M, Rostami K (2015). Non-celiac gluten sensitivity has narrowed the spectrum of irritable bowel syndrome: a double-blind randomized placebo-controlled trial. Nutrients.

[88] Zanini B, Basche R, Ferraresi A, Ricci C, Lanzarotto F, Marullo M, Villanacci V, Hidalgo A, Lanzini A (2015). Randomised clinical study: gluten challenge induces symptom recurrence in only a minority of patients who meet clinical criteria for non-coeliac gluten sensitivity. Aliment Pharmacol Ther.

[89] Zanwar VG, Pawar SV, Gambhire PA, Jain SS, Surude RG, Shah VB, Contractor QQ, Rathi PM (2016). Symptomatic improvement with gluten restriction in irritable bowel syndrome: a prospective, randomized, double blinded placebo controlled trial. Intest Res.

[90] Elli L, Tomba C, Branchi F, Roncoroni L, Lombardo V, Bardella MT, Ferretti F, Conte D, Valiante F, Fini L (2016). Evidence for the presence of non-celiac gluten sensitivity in patients with functional gastrointestinal symptoms: results from a multicenter randomized double-blind placebo-controlled gluten challenge. Nutrients.

[91] Skodje GI, Henriksen C, Salte T, Drivenes T, Toleikyte I, Lovik AM, Veierod MB, Lundin KE (2017). Wheat challenge in self-reported gluten sensitivity: a comparison of scoring methods. Scand J Gastroenterol.

[92] Dale HF, Hatlebakk JG, Hovdenak N, Ystad SO, Lied GA (2018). The effect of a controlled gluten challenge in a group of patients with suspected non-coeliac gluten sensitivity: a randomized, double-blind placebo-controlled challenge. Neurogastroenterol Motil.

[93] Kaukinen K, Turjanmaa K, Maki M, Partanen J, Venalainen R, Reunala T, Collin P (2000). Intolerance to cereals is not specific for coeliac disease. Scand J Gastroenterol.

[94] Pinto-Sanchez MI, Verdu EF (2018). Non-celiac gluten or wheat sensitivity: it's complicated!. Neurogastroenterol Motil.

[95] Tanpowpong P, Ingham TR, Lampshire PK, Kirchberg FF, Epton MJ, Crane J, Camargo CA, New Zealand A (2012). Allergy Cohort Study G: coeliac disease and gluten avoidance in New Zealand children. Arch Dis Child.

[96] Dale HF, Biesiekierski JR, Lied GA (2019). Non-coeliac gluten sensitivity and the spectrum of gluten-related disorders: an updated overview. Nutr Res Rev.

[97] Fritscher-Ravens A, Pflaum T, Mosinger M, Ruchay Z, Rocken C, Milla PJ, Das M, Bottner M, Wedel T, Schuppan D (2019). Many patients with irritable bowel syndrome have atypical food allergies not associated with immunoglobulin E. Gastroenterology.

[98] Sergi C, Shen F, Bouma G (2017). Intraepithelial lymphocytes, scores, mimickers and challenges in diagnosing gluten-sensitive enteropathy (celiac disease). World J Gastroenterol.

[99] Sapone A, Bai JC, Ciacci C, Dolinsek J, Green PH, Hadjivassiliou M, Kaukinen K, Rostami K, Sanders DS, Schumann M (2012). Spectrum of gluten-related disorders: consensus on new nomenclature and classification. BMC Med.

[100] Ludvigsson JF, Leffler DA, Bai JC, Biagi F, Fasano A, Green PH, Hadjivassiliou M, Kaukinen K, Kelly CP, Leonard JN (2013). The Oslo definitions for coeliac disease and related terms. Gut.

[101] Armstrong D, Don-Wauchope AC, Verdu EF (2011). Testing for gluten-related disorders in clinical practice: the role of serology in managing the spectrum of gluten sensitivity. Can J Gastroenterol.

[102] Hammond C, Lieberman JA (2018). Unproven diagnostic tests for food allergy. Immunol Allergy Clin North Am.

[103] Hayat M, Cairns A, Dixon MF, O'Mahony S (2002). Quantitation of intraepithelial lymphocytes in human duodenum: what is normal?. J Clin Pathol.

[104] Veress B, Franzen L, Bodin L, Borch K (2004). Duodenal intraepithelial lymphocyte-count revisited. Scand J Gastroenterol.

[105] Hudacko R, Kathy Zhou X, Yantiss RK (2013). Immunohistochemical stains for CD3 and CD8 do not improve detection of gluten-sensitive enteropathy in duodenal biopsies. Mod Pathol.

[106] Mubarak A, Wolters VM, Houwen RH, ten Kate FJ (2015). Immunohistochemical CD3 staining detects additional patients with celiac disease. World J Gastroenterol.

[107] Memeo L, Jhang J, Hibshoosh H, Green PH, Rotterdam H, Bhagat G (2005). Duodenal intraepithelial lymphocytosis with normal villous architecture: common occurrence in H. pylori gastritis. Mod Pathol.

[108] Brown I, Mino-Kenudson M, Deshpande V, Lauwers GY (2006). Intraepithelial lymphocytosis in architecturally preserved proximal small intestinal mucosa: an increasing diagnostic problem with a wide differential diagnosis. Arch Pathol Lab Med.

[109] Sergi C, Lam J, Persad R (2020). Clostridium ventriculi infection in a child with phenylketonuria. Ann Clin Lab Sci.

[110] Villanacci V, Lanzini A, Lanzarotto F, Ricci C (2013). Observations on the paper of Carroccio et al. "non-celiac wheat sensitivity diagnosed by double-blind placebo-controlled challenge: exploring a new clinical entity". Am J Gastroenterol.

[111] Zanini B, Villanacci V, Marullo M, Cadei M, Lanzarotto F, Bozzola A, Ricci C (2018). Duodenal histological features in suspected non-celiac gluten sensitivity: new insights into a still undefined condition. Virchows Arch.

[112] Carroccio A, Giannone G, Mansueto P, Soresi M, La Blasca F, Fayer F, Iacobucci R, Porcasi R, Catalano T, Geraci G (2019). Duodenal and rectal mucosa inflammation in patients with non-celiac wheat sensitivity. Clin Gastroenterol Hepatol.

[113] Sapone A, Lammers KM, Mazzarella G, Mikhailenko I, Carteni M, Casolaro V, Fasano A (2010). Differential mucosal IL-17 expression in two gliadin-induced disorders: gluten sensitivity and the autoimmune enteropathy celiac disease. Int Arch Allergy Immunol.

[114] Verdu EF, Armstrong D, Murray JA (2009). Between celiac disease and irritable bowel syndrome: the "no man's land" of gluten sensitivity. Am J Gastroenterol.

[115] Verdu EF, Huang X, Natividad J, Lu J, Blennerhassett PA, David CS, McKay DM, Murray JA (2008). Gliadin-dependent neuromuscular and epithelial secretory responses in gluten-sensitive HLA-DQ8 transgenic mice. Am J Physiol Gastrointest Liver Physiol.

[116] Caio G, Riegler G, Patturelli M, Facchiano A (2017). L DEM, Sapone A: Pathophysiology of non-celiac gluten sensitivity: where are we now?. Minerva Gastroenterol Dietol.

[117] Sapone A, Lammers KM, Casolaro V, Cammarota M, Giuliano MT, De Rosa M, Stefanile R, Mazzarella G, Tolone C, Russo MI (2011). Divergence of gut permeability and mucosal immune gene expression in two gluten-associated conditions: celiac disease and gluten sensitivity. BMC Med.

[118] Dupont FM, Vensel WH, Tanaka CK, Hurkman WJ, Altenbach SB (2011). Deciphering the complexities of the wheat flour proteome using quantitative two-dimensional electrophoresis, three proteases and tandem mass spectrometry. Proteome Sci.

[119] Santin I, Castellanos-Rubio A, Hualde I, Castano L, Vitoria JC, Bilbao JR (2007). Toll-like receptor 4 (TLR4) gene polymorphisms in celiac disease. Tissue Antigens.

[120] Ziegler K, Neumann J, Liu F, Frohlich-Nowoisky J, Cremer C, Saloga J, Reinmuth-Selzle K, Poschl U, Schuppan D, Bellinghausen I (2018). Nitration of wheat amylase trypsin inhibitors increases their innate and adaptive immunostimulatory potential in vitro. Front Immunol.

[121] Schuppan D, Zevallos V (2015). Wheat amylase trypsin inhibitors as nutritional activators of innate immunity. Dig Dis.

[122] Zheng M, Ambesi A, McKeown-Longo PJ (2020). Role of TLR4 receptor complex in the regulation of the innate immune response by Fibronectin. Cells.

[123] DiGiacomo DV, Tennyson CA, Green PH, Demmer RT (2013). Prevalence of gluten-free diet adherence among individuals without celiac disease in the USA: results from the Continuous National Health and Nutrition Examination Survey 2009–2010. Scand J Gastroenterol.

[124] Aziz I, Lewis NR, Hadjivassiliou M, Winfield SN, Rugg N, Kelsall A, Newrick L, Sanders DS (2014). A UK study assessing the population prevalence of self-reported gluten sensitivity and referral characteristics to secondary care. Eur J Gastroenterol Hepatol.

[125] Aziz I, Hadjivassiliou M (2014). Coeliac disease: noncoeliac gluten sensitivity–food for thought. Nat Rev Gastroenterol Hepatol.

[126] Butterworth JR, Banfield LM, Iqbal TH, Cooper BT (2004). Factors relating to compliance with a gluten-free diet in patients with coeliac disease: comparison of white Caucasian and South Asian patients. Clin Nutr.

[127] Carroccio A, Soresi M, D'Alcamo A, Sciume C, Iacono G, Geraci G, Brusca I, Seidita A, Adragna F, Carta M (2014). Risk of low bone mineral density and low body mass index in patients with non-celiac wheat-sensitivity: a prospective observation study. BMC Med.

[128] Bushara KO (2005). Neurologic presentation of celiac disease. Gastroenterology.

[129] Lionetti E, Francavilla R, Pavone P, Pavone L, Francavilla T, Pulvirenti A, Giugno R, Ruggieri M (2010). The neurology of coeliac disease in childhood: what is the evidence? A systematic review and meta-analysis. Dev Med Child Neurol.

[130] Batista IC, Gandolfi L, Nobrega YK, Almeida RC, Almeida LM, Campos Junior D, Pratesi R (2012). Autism spectrum disorder and celiac disease: no evidence for a link. Arq Neuropsiquiatr.

[131] Buie T (2013). The relationship of autism and gluten. Clin Ther.

[132] Cruchet S, Lucero Y, Cornejo V (2016). Truths, myths and needs of special diets: attention-deficit/hyperactivity disorder, autism, non-celiac gluten sensitivity, and vegetarianism. Ann Nutr Metab.

[133] Casella G, Pozzi R, Cigognetti M, Bachetti F, Torti G, Cadei M, Villanacci V, Baldini V, Bassotti G (2017). Mood disorders and non-celiac gluten sensitivity. Minerva Gastroenterol Dietol.

[134] Slim M, Rico-Villademoros F, Calandre EP (2018). Psychiatric comorbidity in children and adults with gluten-related disorders: a narrative review. Nutrients.

[135] Campisi G, Di Liberto C, Carroccio A, Compilato D, Iacono G, Procaccini M, Di Fede G, Lo Muzio L, Craxi A, Catassi C (2008). Coeliac disease: oral ulcer prevalence, assessment of risk and association with gluten-free diet in children. Dig Liver Dis.

[136] Guandalini S, Polanco I (2015). Nonceliac gluten sensitivity or wheat intolerance syndrome?. J Pediatr.

[137] Tomasello G, Mazzola M, Leone A, Sinagra E, Zummo G, Farina F, Damiani P, Cappello F, Gerges Geagea A, Jurjus A (2016). Nutrition, oxidative stress and intestinal dysbiosis: influence of diet on gut microbiota in inflammatory bowel diseases. Biomed Pap Med Fac Univ Palacky Olomouc Czech Repub.

[138] Joseph J, Depp C, Shih PB, Cadenhead KS, Schmid-Schonbein G (2017). Modified mediterranean diet for enrichment of short chain fatty acids: potential adjunctive therapeutic to target immune and metabolic dysfunction in schizophrenia?. Front Neurosci.

[139] Serra N, Di Carlo P, Gulotta G, d’ Arpa F, Giammanco A, Colomba C, Melfa G, Fasciana T, Sergi C (2018). Bactibilia in women affected with diseases of the biliary tract and pancreas A STROBE guidelines-adherent cross-sectional study in Southern Italy. J Med Microbiol.

[140] Ma G (2015). Food, eating behavior, and culture in Chinese society. J Ethn Foods.

[141] Lucas A, Murray E, Kinra S (2013). Heath beliefs of UK South Asians related to lifestyle diseases: a review of qualitative literature. J Obes.

[142] Probst YC, Peng Q (2018). Social media in dietetics: insights into use and user networks. Nutr Diet.

[143] Stanford FC, Tauqeer Z, Kyle TK (2018). Media and its influence on obesity. Curr Obes Rep.

[144] Das JK, Lassi ZS, Hoodbhoy Z, Salam RA (2018). Nutrition for the next generation: older children and adolescents. Ann Nutr Metab.

[145] Katz DL, Frates EP, Bonnet JP, Gupta SK, Vartiainen E, Carmona RH (2018). Lifestyle as medicine: the case for a true health initiative. Am J Health Promot.

[146] Haghighian Roudsari A, Vedadhir A, Amiri P, Kalantari N, Omidvar N, Eini-Zinab H, Hani Sadati SM (2017). Psycho-socio-cultural determinants of food choice: a qualitative study on adults in social and cultural context of Iran. Iran J Psychiatry.

